# Renal Oxidative Stress Induced by Long-Term Hyperuricemia Alters Mitochondrial Function and Maintains Systemic Hypertension

**DOI:** 10.1155/2015/535686

**Published:** 2015-03-31

**Authors:** Magdalena Cristóbal-García, Fernando E. García-Arroyo, Edilia Tapia, Horacio Osorio, Abraham S. Arellano-Buendía, Magdalena Madero, Bernardo Rodríguez-Iturbe, José Pedraza-Chaverrí, Francisco Correa, Cecilia Zazueta, Richard J. Johnson, Laura-Gabriela Sánchez Lozada

**Affiliations:** ^1^Department of Nephrology, INC Ignacio Chávez, 14080 Mexico City, DF, Mexico; ^2^Laboratory of Renal Physiopathology, INC Ignacio Chávez, 14080 Mexico City, DF, Mexico; ^3^Division of Nephrology, Hospital Universitario de Maracaibo and Laboratory of Immunobiology, Instituto Venezolano Investigaciones Científicas, Maracaibo 04011, Zulia, Venezuela; ^4^Department of Biology, Facultad de Química UNAM, 04510 Mexico City, DF, Mexico; ^5^Department of Cardiovascular Biomedicine, INC Ignacio Chávez, 14080 Mexico City, DF, Mexico; ^6^Division of Renal Diseases and Hypertension, University of Colorado, Denver, CO 80045, USA

## Abstract

We addressed if oxidative stress in the renal cortex plays a role in the induction of hypertension and mitochondrial alterations in hyperuricemia. A second objective was to evaluate whether the long-term treatment with the antioxidant Tempol prevents renal oxidative stress, mitochondrial alterations, and systemic hypertension in this model. Long-term (11-12 weeks) and short-term (3 weeks) effects of oxonic acid induced hyperuricemia were studied in rats (OA, 750 mg/kg BW), OA+Allopurinol (AP, 150 mg/L drinking water), OA+Tempol (T, 15 mg/kg BW), or vehicle. Systolic blood pressure, renal blood flow, and vascular resistance were measured. Tubular damage (urine N-acetyl-*β*-D-glucosaminidase) and oxidative stress markers (lipid and protein oxidation) along with ATP levels were determined in kidney tissue. Oxygen consumption, aconitase activity, and uric acid were evaluated in isolated mitochondria from renal cortex. Short-term hyperuricemia resulted in hypertension without demonstrable renal oxidative stress or mitochondrial dysfunction. Long-term hyperuricemia induced hypertension, renal vasoconstriction, tubular damage, renal cortex oxidative stress, and mitochondrial dysfunction and decreased ATP levels. Treatments with Tempol and allopurinol prevented these alterations. Renal oxidative stress induced by hyperuricemia promoted mitochondrial functional disturbances and decreased ATP content, which represent an additional pathogenic mechanism induced by chronic hyperuricemia. Hyperuricemia-related hypertension occurs before these changes are evident.

## 1. Introduction

Over recent years, epidemiological studies and clinical intervention trials, including randomized controlled trials, have shown that hyperuricemia is likely a cause or exacerbating factor of hypertension and progressive kidney disease [1–3].

Experimental studies demonstrated that experimental hyperuricemic hypertension is associated with inflammation, renal microvascular damage, and renal vasoconstriction [4–8].

Oxidative stress seems to be a primary deletereous effect induced by increased uric acid (UA) [4]. In this regard, the role of the activation of NADPH oxidase by hyperuricemia has been well established [4, 9, 10]. On the other hand, a large fraction of reactive oxygen species (ROS) can be of mitochondrial origin, and mitochondrial abnormalities have also been described during hypertension [11, 12]. We previously reported that UA induced a reduction of mitochondrial mass with concomitant depletion of aconitase (ACO-2) and enoyl CoA hydratase-1 (ECoAH-1) in endothelial cells [13]. In addition, hyperuricemic rats had lower mitochondrial DNA (mtDNA) in the renal cortex in association with higher levels of intrarenal UA and oxidative stress [13].

Since long-term regulation of blood pressure is regulated by kidney, it is important to establish whether hyperuricemia induced hypertension may be a secondary effect of increased renal oxidative stress. Therefore, the first objective of this study was to address if oxidative stress in the renal cortex might play a role in the induction of hypertension and mitochondrial alterations in this model. A second objective was to evaluate whether the long-term treatment with the antioxidant Tempol prevents renal oxidative stress, mitochondrial alterations, and systemic hypertension.

## 2. Methods

All experiments were performed in male Sprague-Dawley rats in accordance with the Mexican Federal Regulation for Animal Experimentation and Care (NOM-062-ZOO-2001) and were approved by Bioethics and Investigation Committees of Instituto Nacional de Cardiología Ignacio Chavez.

### 2.1. Short-Term Study

In order to determine whether oxidative stress plays a role in the development of hyperuricemic hypertension and mitochondrial alterations studies were done in two groups of rats (*n* = 4 each) that received the inhibitor of uricase oxonic acid (OA, 750 mg/kg BW, oral) or vehicle until they developed systemic hypertension (three weeks). In these animals, blood pressure and mitochondrial function, UA content and oxidative stress were evaluated in the renal cortex.

### 2.2. Long-Term Study

In order to evaluate the long-term effect of antioxidant therapy on systemic hypertension, renal function, and mitochondrial respiratory capacity changes induced by hyperuricemia, the following groups of rats were studied (*n* = 12 each): OA (750 mg/kg BW), OA+Tempol (T, 15 mg/kg BW oral), or vehicle for 11-12 weeks. In addition we compared the effect of preventing the raise of UA induced by OA by dosing allopurinol concomitantly in one additional group (AP, 150 mg/L drinking water). At the end of the study, seven rats were sacrificed with pentobarbital; the kidneys were excised and the cortex and medulla surgically separated. The left kidney cortical tissue was used to isolate mitochondria, and the right kidney cortex was snap frozen for additional studies. Five rats from each group were used to determine renal blood flow and vascular resistance.

## 3. Measurements

Systolic blood pressure (SBP) was measured in conscious rats by a validated volume-based tail cuff method [14]. Plasma UA was measured using a commercial kit (DCL Diagnostics, Charlottetown, Canada).

### 3.1. Renal Blood Flow

Rats were anesthetized with sodium pentobarbital (30 mg/kg, i.p.) and placed on a homeothermic table. Rats were maintained under euvolemia by infusion of isotonic bovine serum albumin (BSA, 5 mg/dL) during surgery, followed by an infusion of physiologic saline (0.9%). Mean arterial pressure (MAP) was continuously monitored. An ultrasound flow probe (TS420, Transonic Systems, Ithaca, NY, USA) was placed around the left renal artery to record renal blood flow (RBF). Renal vascular resistance (RVR) and renal plasma flow (RPF) were calculated accordingly to the formulas RVR = MAP/RBF and RPF = RBF × (1 − Hct), respectively.

### 3.2. Mitochondrial Studies

Mitochondria were isolated from the renal cortex by differential centrifugation as previously described [15]. Proteins were measured by the Bradford method.

Mitochondrial oxygen consumption was measured using a Clark-type oxygen electrode (Yellow Springs Instruments, Yellow Springs, OH, USA). State 4 respiration rate was evaluated in 1.5 mL of basic medium containing 125 mM KCl, 10 mM HEPES, 3 mM Pi and 10 mM succinate plus 1 *μ*g/mL rotenone, or 5 mM sodium glutamate plus 5 mM sodium malate. State 3 respiration rate was measured after addition of 200 *μ*M ADP. The respiratory control index (RC) was calculated as the ratio between state 3/state 4 rates.

Aconitase activity was measured in isolated mitochondria as the formation of* cis*-aconitate from isocitrate at 240 nm in Tris-HCl buffer, pH 7.4 in a medium containing isocitrate and MnCl_2_. One unit was defined as the amount of enzyme necessary to produce 1 *μ*mol of* cis*-aconitate/min.

UA was extracted from mitochondria and measured using a commercial kit (DCL Diagnostics, Charlottetown, Canada). Values of UA were normalized by protein concentration.

### 3.3. Oxidative Stress

Tissue was homogenized in phosphate buffer containing a cocktail of proteases inhibitors. Protein carbonyls and 4-hydroxynonenal (4-HNE) were measured using previously published methods [16].

### 3.4. ATP Content

Renal cortical ATP levels were measured by bioluminescence using a commercial kit (ATP Bioluminescence Assay Kit CLS II, Roche Molecular Biochemicals, Mannheim, Germany) accordingly to the manufacturer instructions.

### 3.5. Statistical Analysis

Values are expressed as mean ± standard deviation (SD). For long-term study, significant differences between the three groups were determined by two-way ANOVA. When the ANOVA *P* value was <0.05, posttest comparisons were made using a Bonferroni multiple-comparison test. For the short-term studies, significant differences between OA and control groups were determined by Student's *t*-test. Correlation analysis assessed the relationship between variables. Statistical analysis was performed with Prism version 5.04 (Graph Pad Software, San Diego, CA, USA).

## 4. Results

### 4.1. Short-Term Hyperuricemia Induced Systemic Hypertension but Not Renal Oxidative Stress Neither Mitochondrial Alterations (Figure 1)

**Figure 1:**
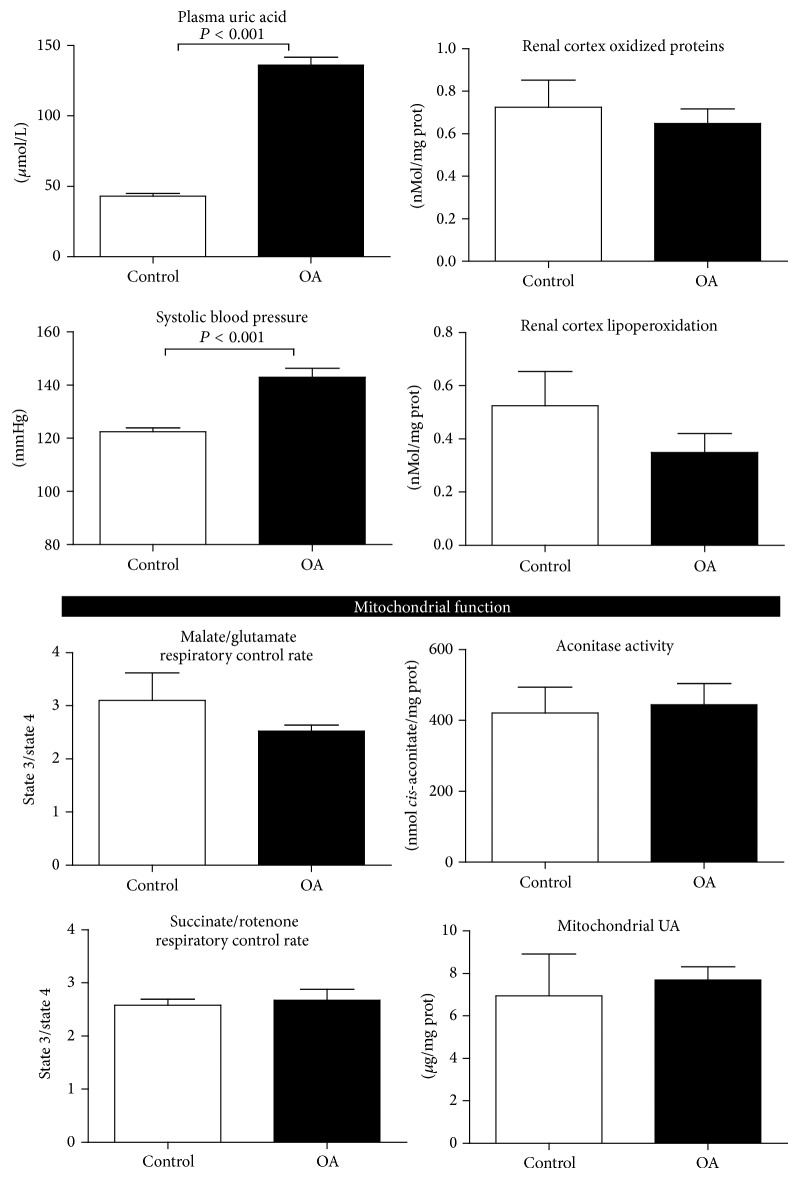
Short-term hyperuricemia induced systemic hypertension but not renal oxidative stress and mitochondrial alterations. Three weeks of oxonic acid dosing induced hyperuricemia and systemic hypertension, but not renal oxidative stress neither renal mitochondrial functional abnormalities.

OA-induced hyperuricemia was associated with the development of systemic hypertension after three weeks of OA dosing (Figure 1). At this time point, mitochondria from these animals showed increased basal respiration rate (state 4) with both NADH linked substrates and with succinate. State 3 respiratory rate was also increased, indicating oxidative phosphorylation coupling irrespective of OA treatment (Figure 1). These results were in agreement with similar activity of mitochondrial aconitase and equivalent UA concentrations in the control and OA-treated group (Figure 1). No changes in oxidative stress markers in the renal cortex were observed in the short-term studies (Figure 1).

### 4.2. Long-Term Hyperuricemia Induced Hypertension, as well as Renal Hemodynamic and Mitochondrial Abnormalities: Antioxidant Treatment Successfully Prevented Those Alterations (Figure 2 and Table 1)

#### 4.2.1. Systemic and Renal Hemodynamics

**Figure 2:**
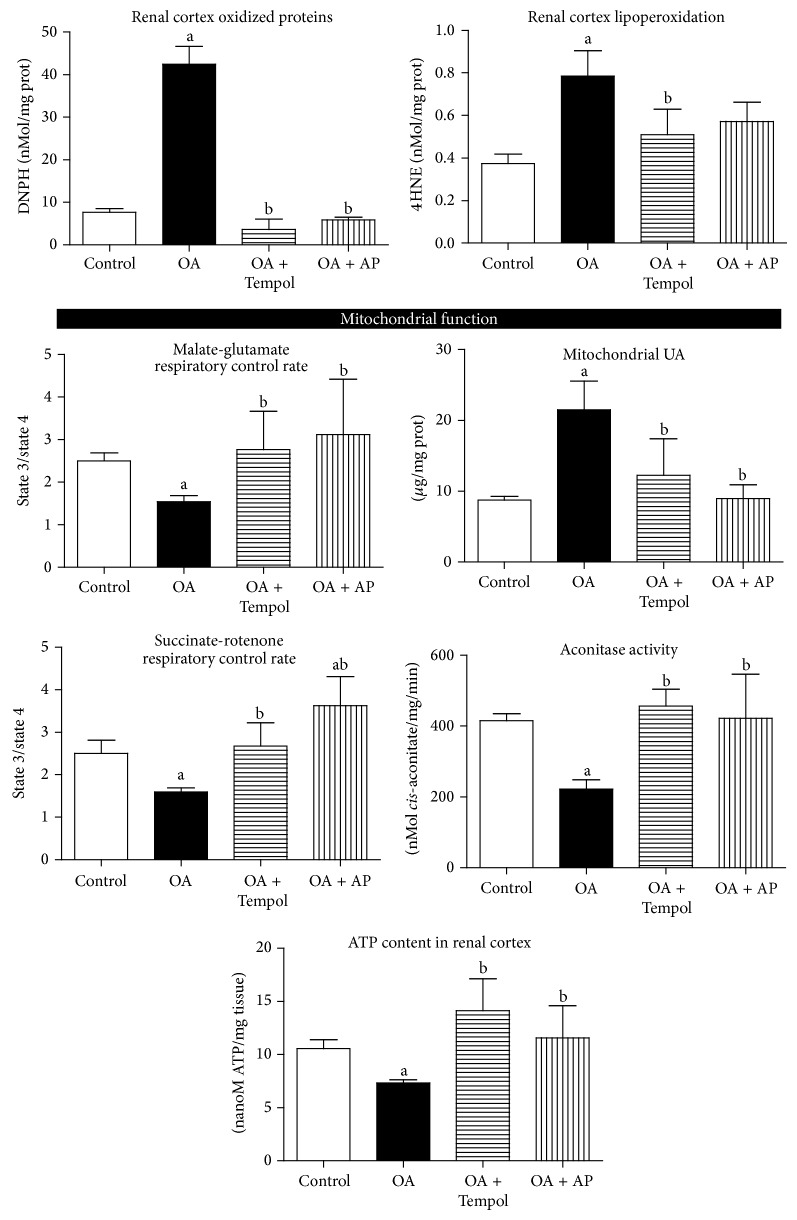
Antioxidant treatment successfully prevented long-term hyperuricemia induced renal biochemical and mitochondrial abnormalities. Long-term OA-induced increased oxidative stress and mitochondrial accumulation of UA. These changes were associated with renal cortical mitochondrial dysfunction characterized by oxidative phosphorylation uncoupling (decreased RC) and decreased aconitase activity and diminished ATP renal content. Tempol and allopurinol treatments prevented those changes. a: *P* < 0.05 versus Control; b: *P* < 0.05 versus OA.

Long-term OA treatment induced hyperuricemia, hypertension, decreased renal blood and plasma flow, and increased renal vascular resistance. Antioxidant treatment with Tempol did not prevent the rise in plasma UA; however, it blocked the development of systemic hypertension as well as renal hemodynamic changes (Figure 2 and Table 1). Allopurinol treatment prevented hyperuricemia, the associated hypertension as well as the renal hemodynamic changes, as previously reported (Table 1 and Figure 1) [7, 8].

#### 4.2.2. Mitochondrial Studies

Mitochondrial respiratory rate coupled to ATP production was depressed in the renal cortex of OA-treated hyperuricemic rats. Treatment with both Tempol and allopurinol maintained mitochondrial respiration similar to control animals. In addition, the activity of aconitase, a marker of superoxide radical damage [17], was significantly reduced in hyperuricemic rats and was preserved in normal levels in the Tempol and allopurinol treated rats. Finally, mitochondrial UA accumulation was induced by OA treatment; this effect was fully prevented by Tempol and allopurinol treatments (Table 1 and Figure 2).

Mitochondrial UA was negatively correlated with aconitase activity (*P* < 0.01).

#### 4.2.3. Markers of Oxidative Stress and ATP Levels in the Renal Cortex

OA-treated rats had increased lipid peroxidation and carbonyl protein oxidation products. Tempol and allopurinol treatments prevented the oxidative stress induced by hyperuricemia. Accordingly, ATP content was reduced in the renal cortex of hyperuricemic animals. Tempol and allopurinol treatments preserved the concentration of renal cortical tissue ATP in similar values as control rats.

## 5. Discussion

Long-term hypertension is a defect intricately associated with kidney functional and/or structural alterations. On the other hand, hyperuricemia has been described as a risk factor for the development of systemic hypertension and chronic kidney disease [1–3]. A primary mechanism involved in those uric acid-mediated effects is a significant increment in oxidative stress [4, 13]. Another potential, as well as poorly explored mechanism of damage induced by hyperuricemia, is an abnormal mitochondrial function. Therefore, these studies were designed to shed more light on the participation of renal oxidative stress as a causal mechanism to induce hyperuricemic hypertension. In addition, we evaluated the treatment with an antioxidant in long-term hyperuricemia and performed functional studies in renal cortex isolated mitochondria in order to define its potential role as an additional injurious effect of hyperuricemia.

Three weeks were required for hyperuricemia to induce hypertension in rats. At this time point, we did not find evidence of increased renal cortical oxidative stress in hyperuricemic animals. Thus, these data suggest that the establishment of systemic hypertension during hyperuricemia is an event independent of renal oxidative stress. The mechanisms involved during this initial phase are likely related to a decreased systemic nitric oxide bioavailability [5].

In short-term hyperuricemia, mitochondrial oxygen consumption, aconitase activity, and uric acid were not different between renal cortex mitochondria isolated from oxonic acid treated animals and those isolated from control group. Normal renal cortex mitochondria acutely exposed to uric acid showed alterations neither in mitochondrial respiration nor in aconitase activity (data not shown). Therefore, these data suggest that mitochondrial functional alterations are not an early manifestation of hyperuricemia pathogenesis in kidney.

Nevertheless, some mitochondrial functional abnormalities induced by uric acid have been described in endothelial cultured cells [18]. Previously we showed increased mtDNA damage and oxidative stress induced by long-term hyperuricemia in renal cortical tissue [8]. However, it is not known whether mitochondrial functional status is altered in the kidney of long-term hyperuricemic rats, nor if such effects can be modulated by oxidative stress. Therefore, a second objective of these studies was to evaluate whether the treatment with the antioxidant Tempol can provide a benefit on mitochondrial oxygen consumption and aconitase activity after long-term hyperuricemia, as all of these functions are particularly sensitive to reactive oxygen species induced injury [14]. We also compared Tempol effects with those provided by allopurinol, a drug that blocks the development of hyperuricemia induced by oxonic acid in rats, as well as the hypertension and renal alterations related to increased uric acid [8, 19].

In the present studies, we observed that Tempol and allopurinol provided similar therapeutic benefits in long-term hyperuricemia (11-12 weeks); allopurinol effects likely were related to its antihyperuricemic effect [7, 8]; in contrast, Tempol did not block oxonic acid-induced hyperuricemia but prevented the increment in blood pressure, intrarenal oxidative stress, and the renal hemodynamic alterations associated with increased levels of uric acid. Previously we reported similar effects of Tempol in animals exposed to the effects of hyperuricemia for a shorter term (5 weeks) [4].

On the other hand, the maintenance of intrarenal antioxidant systems is fundamental to preserve kidney function and structure. In this regard, physiological concentrations of renal dopamine have protective effects on oxidative stress in the kidney. Moreover, dopamine acting on its receptors D1R, D2R, and D5R inhibits NADPH oxidase activity and ROS production and also stimulates antioxidant enzymes such as SOD, glutathione peroxidase, glutamyl cysteine transferase, and HO-1, among others [20]. It is unknown whether uric acid alters renal dopamine system; however, there is some evidence of an interaction between them [21].

After long-term hyperuricemia, renal mitochondrial functional alterations were noticed; basal respiration rate (state 4) was not different among the groups with NADH linked substrates (malate/glutamate) or with succinate. However, respiration coupled to ATP production (state 3) was significantly reduced in renal cortical mitochondria of OA-treated animals with either malate/glutamate or succinate. Therefore, reduced values of respiratory control rates were indicative of oxidative phosphorylation uncoupling. Tempol and allopurinol treatments prevented mitochondrial damage, as respiratory control rates were preserved with values similar to control rats with malate/glutamate and with succinate. Mitochondrial aconitase activity was also evaluated, as its inactivation is a marker of superoxide-mediated mitochondrial damage. Aconitase activity in OA-treated rats was significantly lower than that in control, OA+AP and OA+Tempol. In addition, we found that mitochondrial lysates from OA-treated animals had significantly higher uric acid, and this effect was prevented with allopurinol treatment. Interestingly Tempol also prevented mitochondrial uric acid accumulation. Mitochondrial uric acid results from at least three mechanisms: mitochondrial xanthine oxidase activity [22], mitochondrial purine catabolism [23], and uptake from cytoplasmic space [24]. Regulation of systemic and intracellular uric acid levels is a complex process that requires the participation of several different transporters for its uptake and its efflux. Therefore, dysregulation of such equilibrium may alter intra- and extracellular concentrations of UA. Plant-derived substances with antioxidant activity such as quercetin [25] and nuciferine [26] prevented hyperuricemia induced by oxonic acid in mice by altering the expression and activity of various uric acid transporters. It has been shown that, in order to exert deleterious effects, UA depends on its intracellular concentration. In this context, the regulation of UA transporters is of utmost importance. Verzola et al. showed that URAT1 is fundamental for UA to enter into proximal tubular cells and induce intracellular oxidative stress [27]. In the present studies, we found that Tempol treatment had a beneficial effect despite the fact that it did not reduce UA plasma levels. These results suggest that Tempol might also differentially regulate UA transporters, overall resulting in decreased levels of intracellular UA, thus conferring protection. In support to this contention, we found that Tempol was able to prevent intramitochondrial increment of UA.

Since we found diminished respiration coupled to ATP production with long-term oxonic acid-induced hyperuricemia, we also quantified ATP concentration in cortical tissue of the various groups. Chronic hyperuricemia induced a significant reduction of ATP content in renal cortex, which was similar to our previous findings in endothelial cells [13]. This effect was prevented by the treatment with Tempol and partially with allopurinol, therefore suggesting that preservation of mitochondrial respiration coupling may contribute to maintaining ATP renal content.

We have previously shown that chronic hyperuricemia induces glomerular hypertension and renal cortical vasoconstriction [8, 19]. In the present studies, we directly measured renal blood flow and confirmed a reduction in this parameter [8, 19]. Tempol and allopurinol treatments successfully prevented all these renal functional alterations.

In summary, in the establishment of hyperuricemic hypertension, there is a dissociation of renal oxidative stress and hypertension that likely reflects an intact intrarenal antioxidant system at that early time point. On the other hand, during long-term hyperuricemia, renal oxidative stress is a key mechanism for sustaining systemic hypertension, which is a physiologic response to persistent renal vasoconstriction. In addition, the present studies suggest that renal oxidative stress induced by hyperuricemia likely promotes mitochondrial functional disturbances and decreased ATP content, which represent an additional pathogenic mechanism induced by chronic hyperuricemia.

## Figures and Tables

**Table 1:** Functional and mitochondria data in long-term follow-up groups.

Parameter	Vehicle	OA	OA + Tempol	OA + AP
Plasma UA (*μ*mol/L)	44 ± 2	110 ± 27^a^	117 ± 36^a^	26 ± 6^b^
Systolic blood pressure (mmHg)	125 ± 4	144 ± 7^a^	121 ± 5^b^	129 ± 8^b^
RPF (mL/min)	3.7 ± 0.3	2.7 ± 0.4^a^	4.4 ± 0.3^ab^	4.1 ± 0.3^b^
RVR (mmHg/mL/min)	19 ± 1	28 ± 5^a^	15 ± 1^b^	17 ± 3^b^

	Mitochondrial respiration malate/glutamate			
State 3 (ng AtO_2_/min/mg prot)	83 ± 18	43 ± 13^a^	149 ± 44^ab^	114 ± 43^b^
State 4 (ng AtO_2_/min/mg prot)	34 ± 6^c^	30 ± 11^c^	57 ± 21	37 ± 5^c^

	Mitochondrial respiration succinate/rotenone			
State 3 (ng AtO_2_/min/mg prot)	170 ± 60	84 ± 36^a^	238 ± 53^b^	237 ± 60^b^
State 4 (ng AtO_2_/min/mg prot)	70 ± 20	53 ± 25	87 ± 20^b^	65 ± 14

Data are expressed as mean ± SD. RPF = renal plasma flow; RVR = renal vascular resistance. ^a^ *P* < 0.05 versus control; ^b^ *P* < 0.05 versus OA; ^c^ *P* < 0.05 versus Tempol.
